# Motivational determinants of physical activity in disadvantaged populations with (pre)diabetes: a cross-cultural comparison

**DOI:** 10.1186/s12889-022-12539-9

**Published:** 2022-01-24

**Authors:** Jeroen De Man, Francis Xavier Kasujja, Peter Delobelle, Kristi Sidney Annerstedt, Helle Mölsted Alvesson, Pilvikki Absetz, Edwin Wouters, Meena Daivadanam, David Guwatudde, Thandi Puoane, Roy Remmen, Hanani Tabana, Josefien Van Olmen

**Affiliations:** 1 Department of Family Medicine and Population Health, Centre for General Practice, University of Antwerp, Doornstraat 331, Wilrijk, 2610 Antwerp, Belgium; 2 Department of Epidemiology and Biostatistics, School of Public Health, College of Health Sciences, Makerere University, Kampala, Uganda; 3 Chronic Diseases and Cancer Theme, MRC/UVRI & LSHTM Uganda Research Unit, Entebbe, Uganda; 4 School of Public Health, University of the Western Cape, Belville, South Africa; 5 Chronic Disease Initiative for Africa, University of Cape Town, Cape Town, South Africa; 6 Department of Public Health, Vrije Universiteit Brussel, Brussels, Belgium; 7 Department of Global Public Health, Karolinska Institutet, Stockholm, Sweden; 8 Collaborative Care Systems Finland, Helsinki, Finland; 9 Institute of Public Health and Clinical Nutrition, University of Eastern Finland, Kuopio, Finland; 10 Centre for Population, Family & Health, Department of Sociology, University of Antwerp, Antwerp, Belgium; 11 Department of Women’s and Children’s Health, Uppsala University, Uppsala, Sweden; 12 Department of Food Studies, Nutrition and Dietetics, Uppsala University, Uppsala, Sweden; 13 Department of Public Health, Institute of Tropical Medicine Antwerp, Antwerp, Belgium

**Keywords:** Physical activity, Self-efficacy, Social support, Self-determination theory, Vulnerable populations, South Africa, Uganda, Sweden, Type 2 diabetes, Measurement invariance

## Abstract

**Background:**

Understanding motivational determinants of physical activity (PA) is essential to guide the implementation of PA at individual and population level. Knowledge about the cross-cultural generalizability of these determinants is lacking and they have mostly been studied as separate factors. This study compares a motivational process model across samples from diverse populations with, or at risk of diabetes.

**Methods:**

Measurement invariance of barrier identified regulation, barrier self-efficacy and social support was assessed in a rural Ugandan sample (*n* = 712) and disadvantaged samples with high proportions of immigrants in urban South Africa (*n* = 566) and Sweden (*n* = 147). These motivational determinants were then compared through multigroup structural equation modeling.

**Results:**

The studied motivational constructs showed scalar invariance. Latent mean levels of perceived social support and barrier self-efficacy were lower in South Africa and Sweden. Structural models (for different PA outcomes) were not consistent across settings except for the association between perceived social support and identified regulation. Identified regulation was only associated with vigorous PA in Uganda and with moderate PA in South Africa. The association between social support and PA outcomes ranged from weak to not significant and the association between self-efficacy and PA was not significant. Self-reported PA was highest in Uganda and lowest in Sweden. Self-reported vigorous PA was significantly related to lower hemoglobin A1c levels, while moderate PA was not.

**Conclusions:**

Findings suggest that: 1) it is feasible to compare a motivational process model across diverse settings; 2) there is lower perceived social support and self-efficacy in the urban, migrant samples; 3) identified regulation is a more promising determinant of PA than self-efficacy or social support in these populations; 4) associations between motivational determinants and PA depend on the perceived type and/or intensity of PA; 5) perceived relatedness functions as a basic psychological need across diverse settings; and 6) people’s perception of the PA they perform depends on their perceived level of intensity of PA which would have major implications for health promotion.

**Supplementary Information:**

The online version contains supplementary material available at 10.1186/s12889-022-12539-9.

## Background

Type 2 diabetes (T2D) is known as one of the globe’s top killers and causes of disability [1]. Sub-Saharan Africa, in particular, is to experience the highest increase in prevalence by 2045 of all global regions [1]. In high-income countries, socioeconomically disadvantaged communities have been disproportionally affected [2]. The beneficial role of physical activity (PA) in the prevention of T2D has been well established [3] and more people engaging in regular PA could help in curbing this growing pandemic. For low-income sub-Saharan African countries, implementation of PA is a feasible prevention strategy.

Incorporating regular PA in people’s daily lives, however, remains a challenge. Sustainable behavior change is known to be complicated and dependent on many factors including a supportive physical and social environment [4]. Behavioral theories have been shown useful in the adoption of regular PA, but most evidence to support such theories has originated from populations that are Western, educated, industrialized, rich and democratic (WEIRD), and as such, have been called “frequent outliers” – i.e., the least representative populations in terms of human psychology and behavior [5]. Cross-sociocultural validation of behavioral theories among non-WEIRD populations is therefore urgently needed.

Self-Determination Theory (SDT) has offered promising insights in people’s engagement in sustainable lifestyle behavior such as performing regular PA [6]. SDT argues that the quality of people’s social environment plays a crucial role in maintaining such behavior. SDT distinguishes between *autonomous* forms of motivation which emanate from within oneself or from abiding values and *controlled* forms of motivation which are triggered by sources external to the actual behavior [7]. Social contexts satisfying an individual’s perceived competence, autonomy, and relatedness (defined as the basic psychological needs) have been shown to foster more autonomous types of motivation resulting in a more sustained behavior change [7].

SDT has been claimed to be etic universal, meaning that its cross-cultural validity can be empirically identified [7]. However, existing studies on cross-cultural generalizability have been focused on comparing industrialized countries in high- and middle-income regions [8]. And, to our knowledge, no studies comparing SDT’s generalizability between sub-Saharan Africa and other settings have been published yet. Another limitation is that most of the existing studies on cross-cultural generalizability have focused on specific domains such as education, work and well-being [9–11]. While evidence supports SDT with regards to engagement in sustained PA [6], cross-cultural validation has been scarce and limited to students or athletes [12–14].

A similar conclusion can be drawn for social support and self-efficacy, two constructs that have been consistently linked to PA, including in low- and middle income settings [15, 16]. Evidence for these associations, however, typically comes from single country analyses which may not guarantee cross-cultural generalizability. Moreover, studies have typically zoomed in on one of both factors, ignoring the possible interactions between concepts.

To address this last shortcoming, we recently assessed an SDT-based process model with integration of social support and self-efficacy and with PA as the intended behavior in a rural Ugandan population [17]. The study showed a positive relationship between the frequency of vigorous PA and identified regulation, a form of autonomous motivation elicited through associating PA with an individuals’ goals or values, such as “being healthy”. Further in line with SDT, identified regulation operated as a mediator between vigorous PA and barrier self-efficacy and perceived social support, which show conceptual and statistical parallels with, respectively, perceived competence (i.e. one’s sense of efficacy with respect to both internal and external environments) and perceived relatedness (i.e. the sense of being supported by significant others in one’s actions) which we defined earlier as the psychological needs in SDT [7, 17–19]. While this study provided evidence on SDT and PA in sub-Saharan Africa, cross-cultural validation of the theory requires explicit comparison between different settings.

The objective of this study is to compare an adapted version of this process model across socio-economically disadvantaged populations in two sub-Saharan African and one European country, using state-of-the-art modeling techniques. First, we will test if it is feasible to compare motivational determinants of PA (i.e. autonomous motivation, self-efficacy and social support) across diverse socio-cultural environments. Second, we will compare mean levels of these determinants across the three settings. Third, we will test the hypothesized motivational process model in each of the three settings and across the settings. Finally, we will investigate the relationship between PA and HbA1C in a separate model with the aim to connect the self-reported PA outcomes with a more objective measure.

**Fig. 1:**
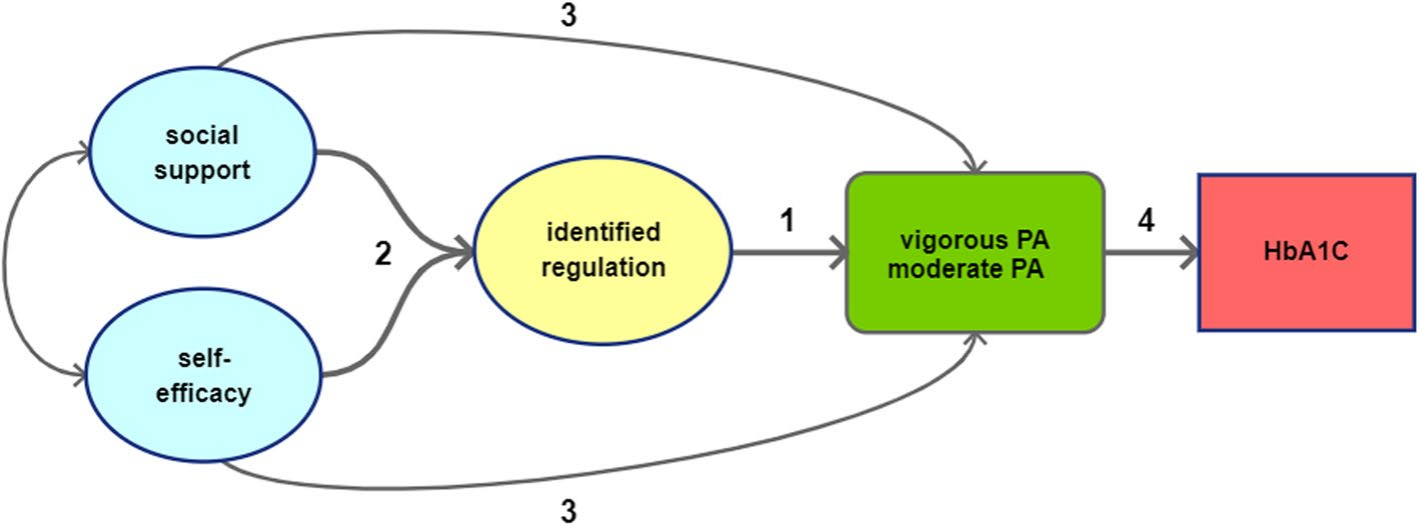
Motivational Process Model. *Legend:* Representation of the motivational process model that was tested in the three study settings. Numbers relate to the hypothesis discussed in the text

The hypothesized model for this study (see Fig. 1), assumes: (1) a positive association between identified regulation (i.e. a form of autonomous motivation) and PA; (2) a positive association between perceived social support, self-efficacy and identified regulation; (3) a positive total effect of perceived relatedness and self-efficacy on PA outcomes (which includes the associations in the previous steps); and (4) a positive association between PA and HbA1c.

## Methods

### Study design and procedures

This study contributes to the validation of a theory-driven framework that guided the implementation of the SMART2D trial (an adaptive implementation trial to improve self-management and to promote a healthy lifestyle among people at risk of or living with T2D in Uganda and South Africa and a feasibility implementation trial in Sweden) [4]. The study used cross-sectional baseline data collected from two rural districts in eastern Uganda, a peri-urban township in the Western Cape in South Africa, and two socio-economically disadvantaged districts of Stockholm in Sweden.

### Study settings

The rural Ugandan population was characterized by a collectivist society with low levels of migration and a high proportion working in agriculture. The urban South African population was characterized by national and international migrant workers with a relatively high unemployment rate. Participants of this setting reported that frequent migration hindered them to build strong community ties. The urban Swedish population consisted of a high proportion of immigrants (approx. 60%) with a diversity in culture and ethnic background living in a society where health and lifestyle are individualized. All three populations were socio-economically disadvantaged in several aspects, but with a sharper socio-economic inequality in the South African setting. More details about the social and built environment, the health system and the population of the study sites can be found elsewhere [4].

### Study participants, sampling and recruitment

Study participants were considered eligible if they had resided in one of the study sites for at least 6 months; were aged 30–75 years; had not been previously diagnosed with T2D for longer than 12 months (for the Ugandan and South African site) or 5 years (for the Swedish site); and had a confirmation of prediabetes or diabetes. Pregnancy and serious mental disability were exclusion criteria. In Uganda, 712 participants were recruited by trained field research assistants approaching households in the study area in a random manner. In South Africa, 566 participants were recruited from two community health centers located in the township upon referral by a health care worker. In Sweden, 147 participants were recruited through screening in public spaces and facility-based screening in two primary health centers. Consenting participants were screened through a fasting plasma glucose test in Uganda, a random plasma glucose test in South Africa and the Finnish Diabetes Risk Score (FINDRISC) in Sweden, except for diabetes patients recruited directly from the health facility in the Swedish setting. Confirmation of T2D or pre-T2D was done through fasting plasma glucose tests in Uganda and South Africa (at least two tests ≥6.1 mmol/L for pre-T2D and at least two tests ≥6.9 mmol/L for T2D) and through an HbA1c test in Sweden (HbA1c ≥ 42 mmol/mol for pre-T2D and HbA1c ≥ 48 mmol/mol for T2D). More details about the selection criteria and the recruitment process can be found elsewhere for the Ugandan setting [17], the South African setting [20] and the Swedish setting [21].

### Data collection

A questionnaire was administered by trained field workers and included socio-demographic items, PA- and motivation-related scales, anthropometric and biochemical measurements. Data were collected between January 2017 through December 2017 in Uganda, between August 2017 and November 2018 in South Africa and between June 2017 and January 2019 in Sweden.

### Measures

*Identified regulation towards physical exercise* was assessed through the Treatment Self-Regulation Questionnaire for people with diabetes. This scale has been widely used to test PA self-regulation and studies have reported adequate reliability [22]. Guided by factor loadings identified in the study by Levesque et al. [22], four items were selected to measure identified regulation (see Additional file 1). Participants responded to each item on a 5-point Likert-type scale ranging from 1 (strongly disagree) to 5 (strongly agree).

To measure *perceived social support,* an adapted version of the scale for participation and involvement of family members and friends in PA was used [23]. This scale has been used and validated in a variety of contexts [23]. Five items of the initial measure were selected based on their presumed cross-cultural adaptability and factor loadings in previous studies (see Additional file 1). Participants responded to each item on a 4-point Likert-type scale ranging from 1 (never) to 4 (more than once a week). Perceived social support shows conceptual parallels with perceived relatedness and the same scale was used by others to measure perceived relatedness [24]. To emphasize the concept of perceived support among the study participants, we introduced the questions with the following statement: *“We want to understand to what extent people close to you (friends, family or relatives) have helped you to do physical activity”*.

Barrier self-efficacy (or self-regulatory efficacy) corresponds to the perceived capability to maintain PA given various conditions or impediments (i.e. barriers). Six items were adapted from the health-specific self-efficacy scale developed by Schwarzer et al. (2007) (see Additional file 1). Barriers included in the original questionnaire were modified to barriers relevant to the study contexts. Participants responded to each item on a 5-point Likert-type scale ranging from 1 (strongly disagree) to 5 (strongly agree). Self-efficacy and perceived competence have shown to be correlated [25], but a conceptual difference needs to be acknowledged [18]. Unlike self-efficacy, perceived competence encompasses the concept of personal effectance, or the perceived need to effect change and attain valued outcomes [18].

PA was measured through: *1) self-reported frequency of vigorous PA; and 2) self-reported frequency of moderate PA*. Initial questions from the World Health Organisation “STEPS” survey [26] were contextually adapted (see Additional file 1). Different measures were chosen since associations with SDT constructs may depend on the intensity of PA [6]. More detail on the motivational and PA measures can be found in a previous study on SDT by De Man et al. [17].

HbA1c was measured using capillary blood samples obtained with a Point-of-care HbA1c Analyzer Cobas b101 (Roche Diagnostics) with the respective test and control reagents.

Sociodemographic characteristics were self-reported based on the categories included in Table 1. Height and weight were measured using standard procedures.

### Contextual adaption

All measures were translated into the local language of the study populations (i.e. Lusoga, Swedish, Arabic, Somali and isiXhosa), and adapted to the context based on inputs from a team of local research assistants. Measures were then back translated to English and adjustments made where necessary to ensure that the meaning of the questions was not lost. Local validity was ensured through piloting in a non-study area, training of data collectors (e.g. through mock interviews), and minimizing inter-interviewer variability.

### Data analysis

The current state-of-the-art approach to compare mean levels and associations of latent constructs across different settings is multigroup structural equation modelling (MGSEM) [27]. A major condition to apply this technique is measurement invariance of constructs across different settings [27]. Measurement invariance supports the idea that subjects of different subgroups have a similar understanding and give a similar meaning to the items of a latent construct. Testing for measurement invariance was based on subsequent steps imposing additional constraints to the models. Before testing for measurement invariance, separate measurement models were assessed for each construct and country separately. Confirmatory factor analysis (CFA) was used to assess the loadings of the item indicators on the latent variables (i.e. identified regulation, barrier self-efficacy, and social support) and the goodness of fit of these measurement models. Subsequently, simultaneous analysis of equal form (i.e. configural invariance), equivalence of factor loadings (i.e. metric invariance) and equivalence of intercepts (i.e. scalar invariance) was conducted across countries through MGSEM.

In case these measurement models would yield an acceptable fit and were shown invariant, the fit of the hypothesized structural equation model (SEM) was assessed per country separately. Finally, to test if the associations between the constructs across the three countries were similar, we compared the difference in model fit between a model constraining these associations as similar across settings and a model without constraining a specific association. In case model fit was not significantly worse between these nested models, we concluded that that specific association was similar across settings. Model fit was evaluated based on multiple indices, including root-mean-square error of approximation (RMSEA) corrected for nonnormality [28], with target values as proposed by Hu and Bentler [29]: the comparative fit index (CFI) ≥ 0.95, Tucker-Lewis index (TLI) ≥ 0.95, the root mean square error of approximation (RMSEA) ≤0.06, and the standardized root mean square residual (SRMR) ≤0.08. Criteria indicating a significant decrease in model fit for the more constrained model (i.e. indicating non-invariance) were as follows: a decrease in CFI larger than 0.01 combined with an increase in RMSEA smaller than 0.015 or a non-significant scaled χ-square difference test [30]. Since items’ distributions departed from normality, we used maximum likelihood estimation with robust standard errors and a Satorra-Bentler scaled test statistic [31]. Covariates were added to address potential sources of confounding based on theory and identified through directed acyclic graphs. Education, marital status and body mass index (BMI) were included to adjust the motivational constructs. Age, sex, occupation, BMI and education were included to adjust the PA outcomes [15]. Data were analyzed using R software with the packages “lavaan” and “semTools”. To study the link between PA outcomes and HbA1c, a separate linear regression model was used controlling for the following covariates: age, sex, BMI and reported intake of oral antidiabetic medication [32].

### Missing data

Missing data for the Ugandan site varied from 0.0–1.3% per variable, for the South African site from 0.0–1.4% and 2.0–10.2% for the Swedish site. The variable responding to the question: “are you currently on any oral hypoglycemic agents?” was missing more frequently: 49.4, 51.2 and 14.3% among participants in the Uganda, South African and Swedish site respectively. BMI was missing among 16.3% of the participants in the Swedish site. Multivariate imputation by chained equations with predictive mean matching was used to handle the missing data under a missing at random assumption. Rubin’s rules were used to pool point and SE estimates across 30 imputed data sets. The procedure was done using the “Mice” package in R. For the variable regarding oral hypoglycemic treatment, a sensitivity analysis was ran ignoring this variable.

### Ethics approval

The study was approved by the ethics committees in each of the respective countries (See declarations). Informed consent was obtained from all individual participants included in the study.

## Results

### Description of the study samples

Essential characteristics of the samples are summarized in Table 1. The proportion of females was larger in each of the samples. Educational level, BMI, employment and marital status differed substantially across samples. Educational level was lowest in the Ugandan sample and highest in the Swedish sample. A high level of employment was found in the Ugandan sample, while employment was lower in the Swedish and South African samples. Compared to the Ugandan sample, the distribution of BMI was less favorable in the Swedish (87% > 25 kg/m^2^ & 18% > 35 kg/m^2^) and in the South African sample (90% > 25 kg/m^2^ & 43% > 35 kg/m^2^). Participants in the Ugandan sample scored high in terms of self-reported vigorous PA, while self-reported moderate PA was similar to participants in the South African sample. Participants in the Swedish sample scored lower in terms of moderate PA compared to both other samples. Among the participants of the Swedish sample, 45% were born in Europe and 37% were native Swedes. Other participants of the Swedish sample originated form a variety of countries, mostly located in Africa and the Middle-East.

**Table 1:** Demographics, diabetes parameters and physical activity behavior of the study population per setting

		Uganda^a^	South Africa^b^	Sweden^c^
		*Mean (SD)*		
Age in years		52.5 (10.4)	51.5(10.3)	54.9 (11.6)
HbA1C in %		7.8 (2.6)	7.1 (2.2)	6.2 (.7)
		*Proportion*		
Sex	Female	.66	.73	.60
	Male	.34	.27	.40
Education	No Primary	.50	.11	.02
	Primary	.30	.15	.03
	Secondary	.17	.70	.33
	Higher	.02	.04	.62
Marital Status	Married or Cohabiting	.71	.55	.52
	Other	.29	.45	.48
Employment	Yes	.93	.43	.52
	No	.07	.57	.48
Diagnosis	Diabetes	.53	.50	.40
	At Risk	.47	.50	.60
BMI (kg/m^2^)	< 25	.49	.10	.13
	25–35	.45	.48	.69
	> 35	.05	.43	.18
		*Median (1st, 3rd quartile) of N° of days per week*		
Vigorous PA (≥15 min.)		5 (2,7)	2 (1,3)	0 (0,1.5)
Moderate PA (≥30 min.)		6 (3,7)	7 (3,7)	4 (1,7)

*Legend: HbA1c* Hemoglobin A1c, *PA* Physical activity, *BMI* Body Mass Index. ^a^reported in [17]. ^b^reported in [20], except for HbA1c and PA. ^c^reported in [21], except for HbA1c and PA

### Measurement models and invariance

As mentioned above, we first intended to obtain adequate measurement models per country setting based on the same items. The proposed measures for social support and barrier self-efficacy did not yield an acceptable model fit across the three settings (see Additional file 1). To obtain acceptable measurement models, we considered the models’ modification indices and items’ factor loadings. This resulted in.

exclusion of item 2 for the social support construct (see Additional file 1), exclusion of items 1 & 2 for the barrier self-efficacy construct, and adding correlated errors between item 3 & 4 for identified regulation (the items of this construct were mixed up with other items to minimize acquiescence bias, but items 3 & 4 followed after each other). Despite piloting the questionnaire, some participants reported item 1 of the barrier self-efficacy construct as confusing. The two other excluded items may have been interpreted as more directive than intended in the South African setting (i.e. “being told” instead of “encouraged”). Model fit of the resulting measurement modelswas good to excellent for the three settings (see Table 2). Unstandardized factor loadings were all significant (z > 1.96) and standardized factor loadings were higher than .50 (see Additional file 1), except for barrier self-efficacy. For this construct, two items had very low factor loadings (item 6 λ = .23; *P* = .115 and item 5 λ = .32; *P* = .039) in the Swedish setting. We ran a sensitivity analysis with a construct without item 6 which did not result in major differences in the results to follow. In a second step, multigroup confirmatory factor analysis was conducted across the three settings assessing the difference in fit for different levels of invariance. The three constructs showed configural invariance and metric invariance across settings (see Table 2). Social support and identified regulation showed partial scalar invariance (for each, 1 response item was excluded from constraining equal intercepts). The barrier self-efficacy construct showed full scalar invariance.

**Table 2:** Model fit of motivational constructs per country and measurement invariance across the three settings

	χ2	df	n	CFI	TLI	RMSEA	90% CI RMSEA	SRMR	Δχ2	*p*-value	ΔCFI	ΔRMSEA
**Identified Regulation**												
Uganda	.719	1.000	712	1.000	1.000	.000	.000–.126	.006				
South Africa	.110	1.00	566	1.000	1.000	.000	.000–.083	.002				
Sweden	1.129	1.000	147	1.000	1.000	.000	.000–.249	.018				
Configural	1.527	3.000	1425	1.000	1.000	.000	.000–.060	.004				
Metric	17.315	9.000	1425	1.000	1.000	.000	.000–.071	.033	15.790	.015	.000	.000
Partial scalar item 4^a^	21.094	13.000	1425	1.000	1.000	.000	.000–.058	.034	3.571	.467	.000	.000
**Social Support**												
Uganda	3.024	2.000	712	1.000	1.000	.008	.000–.091	.009				
South Africa	7.049	2.000	566	.997	1.000	.052	.000–.139	.014				
Sweden	3.969	2.000	147	.985	1.000	.078	.000–.204	.035				
Configural	26.405	12.000	1425	.998	1.000	.044	.000–.093	.012				
Metric	14.555	6.000	1425	.995	1.000	.045	.004–.076	.026	11.884	.065	−.003	.001
Partial scalar item 3^a^	42.885	16.000	1425	.990	1.000	.057	.033–.081	.032	16.576	.002	−.005	.012
**Barrier Self-efficacy**												
Uganda	2.199	2.000	712	1.000	1.000	.000	.000–.081	.011				
South Africa	14.233	2.000	566	.982	1.000	.100	.044–.166	.028				
Sweden	2.429	2.000	147	1.000	1.000	.000	.000–.183	.039				
Configural	18.358	6.000	1425	.992	1.000	.060	.014–.103	.017				
Metric	39.333	12.000	1425	.982	1.000	.065	.038–.094	.043	20.953	.002	−.010	.005
Scalar	54.349	18.000	1425	.975	1.000	.062	.040–.085	.047	14.987	.020	−.007	−.003

*Legend: df* degrees of freedom, *CFI* comparative fit index, *TLI* Tucker-Lewis index, *RMSEA* root-mean-square error of approximation, *RMSEA 90% CI* 90% confidence interval for RMSEA, *SRMR* standardized root mean square residual, *Δχ2* difference in χ2, *ΔCFI* difference in CFI, *ΔRMSEA* difference in RMSEA

^a^ indicates the items of which the intercepts were freely estimated

### Comparison of latent means of motivational constructs

The level of invariance established in the previous section (i.e. partial to full scalar invariance) allowed us to compare latent mean estimates across settings. Latent mean estimates were highest in Uganda and lowest in South Africa (see Table 3). The difference in identified regulation between South Africa and Sweden was small. Compared to the other settings, social support was substantially higher in Uganda. Compared to the other settings, barrier self-efficacy was substantially lower in South Africa.

**Table 3:** Latent mean estimates and differences of the motivational constructs

	Identified Regulation			Social Support			Barrier Self-Efficacy		
	Est.	Δ	*P*-value	Est.	Δ	*P*-value	Est.	Δ	*P*-value
Uganda	4.689	.000		2.482	.000		4.065	.000	
South Africa	4.494	−.194	.000	1.978	−.504	.000	3.472	−.593	.000
Sweden	4.552	−.136	.025	2.159	−.323	.001	3.919	−.147	.009
South Africa vs. Sweden		−.058	.371		−.180	.048		−.446	.000

*Legend:* Scales of the estimates correspond to their indicators’ scales; i.e. 1–5 for identified regulation and barrier self-efficacy and 1–4 for social support. *P*-values were produced using the scaled chi-square difference test

### Structural models

The hypothesized structural model was fitted for each country separately. This resulted in excellent model fit in all three settings (Table 4). However, associations differed substantially across settings. Identified regulation was only associated with vigorous PA in Uganda and with moderate PA in South Africa. Social support was associated with PA outcomes in all three settings. Barrier self-efficacy showed to be associated with identified regulation in Uganda, but not in South Africa. In Sweden, we found a positive association which was not significant, possibly due to a relatively small sample size (standard errors in Sweden were 0.252; vs. Uganda 0.035). Social support was associated with both PA outcomes in Uganda, with vigorous PA in South Africa and with moderate PA in Sweden. Barrier self-efficacy was associated with moderate PA, but only in South Africa. In Sweden, we found a positive association which was not significant, again, possibly due to a relatively small sample size (*N* = 147).

**Table 4:** Total effects between motivational constructs and physical activity outcomes

					Uganda^a^		South Africa		Sweden	
					Vigorous PA	Moderate PA	Vigorous PA	Moderate PA	Vigorous PA	Moderate PA
Identified Regulation		→	PA Outcome		1.130***	.378	−.034	1.816***	.067	−.094
Social Support		→	Identified Regulation		.107***	.107***	.101***	.099***	.116*	.112*
Barrier Self-Efficacy		→	Identified Regulation		.167***	.166***	−.057	−.055	.225	.227
Social Support		→	PA Outcome		.327***	.229**	.183*	.068	−.000	.778***
Barrier Self-Efficacy		→	PA Outcome		−.119	.264	−.092	.391**	1.390	1.016
Model fit:		CFI			.952		.922		.929	
		TLI			1.000		1.000		1.000	
		RMSEA			.035		.052		.029	
		90% CI RMSEA			.028–.041		.046–0.059		.000–.049	
		RSMR			.043		.066		.066	

*Legend:* The estimates represent unstandardized coefficients. *PA* physical activity*, CFI* comparative fit index, *TLI* Tucker-Lewis index, *RMSEA* root-mean-square error of approximation, *RMSEA 90% CI* 90% confidence interval for RMSEA, *SRMR* standardized root mean square residual

*p*-value < 0.1 “*”, *p* < 0.05 “**”,*p* < 0.005 “***”. ^a^reported in [17]

Metric invariance was established for all constructs across the three settings which supports a meaningful comparison of associations between these constructs across settings. The same structural model as for the single country settings was used to build a multi-group structural model. Based on the χ2-difference test, this model did result in a significantly worse fit compared to a model in which one of the associations mentioned in Table 4 (e.g. identified regulation and PA) was freely estimated across the three settings, except for the association between social support and identified regulation (for vigorous PA: χ^2^scaled = 2.81; df = 2; *p*-value = .245 and for moderate PA: χ^2^scaled = 2.76; df = 2; *p*-value = .375).

In other words, constraining the latter association as equal across countries did not result in a significantly worse fit, indicating a similar association. The other associations, however, showed to differ across settings.

### Relationship between HbA1c and types of PA

In all three settings, self-reported frequency of vigorous PA (i.e. number of days a week) was negatively associated with HbA1c scores (see Table 5). A regression estimate of − 0.103 corresponds to the reduction in participants’ HbA1c per extra day of self-reported vigorous PA per week. Associations with self-reported frequency of moderate PA were negative, but not significant in all three countries. As mentioned above, the variable regarding oral hypoglycemic agents had a high proportion of missing data. Ignoring this variable did not result in major changes, except for moderate PA in South Africa, where the estimate equaled − 0.067 (*p* = 0.114).

**Table 5:** Association estimates between HbA1c and PA outcomes

	Uganda^a^			South Africa			Sweden		
HbA1c ~	Est	SE	*P*-value	Est	SE	p	Est	SE	*P*-value
VIG PA	−.103	.037	.006	−.075	.046	.099	−.069	.036	.055
VIG PA adj.	−.081	.038	.033	−.089	.045	.051	−.070	.033	.039
MOD PA	−.084	.043	.049	−.068	.042	.107	−.029	.030	.342
MOD PA adj.	−.040	.043	.361	−.039	.054	.463	−.025	.027	.358

*Legend:* Adjusted models control for the covariates as reported in the “Materials and Methods” section. HbA1c is predicted in percentage points. *VIG* vigorous, *MOD* moderate, *PA* physical activity, *adj.* Adjusted, *Est* Estimate, *SE* standard error. ^a^ Reported in [17]; a slight difference is due to different covariates for adjustment

## Discussion

The aim of this study was to compare identified regulation, self-efficacy and social support and their association with PA and HbA1c across divers socio-cultural environments. Comparison of these motivational constructs was possible since measurement invariance could be established across samples, suggesting a similar understanding across study populations. However, obtaining adequate measurement models required modification of the initial measures (e.g., items had to be dropped). Estimates of the motivational constructs were highest in Uganda and lowest in South Africa, with a substantial difference for barrier self-efficacy and social support. Structural models did not correspond across settings. Identified regulation was positively associated with vigorous PA in Uganda and with moderate PA in South Africa. In Sweden, none of the PA outcomes was associated with identified regulation. The strength of the association between social support and identified regulation was similar across settings. Depending on the setting, the association between social support and PA outcomes was weak to insignificant. The association between barrier self-efficacy and PA outcomes was not significant. Self-reported PA was highest in Uganda and lowest in Sweden. Vigorous PA was associated with lower HbA1c across countries, while this association was not significant for moderate PA.

The latent mean level of barrier self-efficacy was much lower in South Africa compared to both other settings, which may have occurred for several reasons. External barriers that are more prevalent in South Africa may hinder people from doing PA, including lack of security in the South African township setting [4]. South Africa’s historical context and pervasive social inequality may also have contributed to the perceived difference in levels of self-efficacy [4]. The lower socioeconomic status of study participants, including many internal migrants, may have affected people’s self-esteem [33], and consequently, their self-efficacy [34]. Potentially, this could also explain the lower self-efficacy level (compared to the Ugandan setting) in the Swedish setting, as the study sample was socioeconomically disadvantaged and included 60% migrants [35].

Social support was perceived to be much higher in the Ugandan setting compared to the other settings. This could be explained by the stronger social ties apparent in the rural Ugandan community versus the urban sample with many migrant workers in the South African and Swedish setting [4]. The proportion of participants that indicated to be married or co-habiting was also substantially higher in Uganda. In qualitative interviews conducted during the formative phase of the SMART2D project, participants from the Swedish setting reported to perform PA on their own, rather than with others, which corresponds to health and lifestyle being more individualized in Sweden [4]. We hope that future research can explore these factors in more depth.

The positive association between identified regulation and PA (vigorous PA in Uganda and moderate PA in South Africa) is in line with previous studies on SDT and PA [6]. However, this association was not found in Sweden, nor for other PA outcomes. We assume this lack of association can be explained by the PA-related questions covering all types of PA, including travel- and work-related PA. It is likely that most of the self-reported PA related to travel and work, especially in the African settings [36], which implies an important effect of triggers external to the activity. The difference between Uganda and South Africa could be explained by a different attitude towards the intensity types of PA, although this hypothesis was not tested in this study. In the rural Ugandan setting, participants may have been more used to performing vigorous activity as 69% of the participants were farmers [17], while in the urban township setting of South Africa, participants reported to perform less vigorous physical activity. As they seem less used to perform vigorous PA, they might perceive it as more demanding and hence, they might be more inclined to connect moderate rather than vigorous PA with autonomous forms of motivation. Other studies have highlighted the importance of the type of PA influencing the relationship between PA and the type of motivation [6]. For example, PA of a more repetitive nature was shown to be stronger associated with identified regulation than with intrinsic motivation [6]. Our study suggests that the association with more autonomous forms of motivation may depend on the perceived intensity of PA and that this association is context dependent.

From a statistical perspective, differences in dispersion of the outcome data may explain why certain associations did or did not occur. For instance, vigorous PA in Sweden (inter-quartile range = 1.5) and in South Africa (inter-quartile range = 2) had a lower dispersion compared to moderate PA in both countries (inter-quartile range = 6 and 4 respectively). In Sweden, 69% of the participants reported to perform 0 days of vigorous PA.

The construct of social support showed a similar positive association with identified regulation across the three country settings. Since this construct shows conceptual parallels with the concept of perceived relatedness [24], this finding supports the etic validity of the basic psychological needs theory, which posits that satisfaction of psychological needs fosters more autonomous forms of motivation and more sustainable behavioral outcomes (Deci and Ryan, 2000). However, this did not apply to the construct of barrier self-efficacy, which showed a positive relationship with identified regulation in Uganda, but not in South Africa and Sweden (although the latter could have been due to a lack of power). Moreover, barrier self-efficacy, which has been shown a consistent predictor of PA [16], did not show an association with PA outcomes. This may be explained by most of PA being related to work or travel, with an important influence by external triggers and not leaving much flexibility to participants. On the other hand, our data did provide support for an association, although small, between social support and self-reported PA. A potential explanation could be that participants included companionship at work or during travel in their conceptualization of social support.

Higher self-reported PA in the African sites of the study compared to a high-income Western country such as Sweden is in line with global trends and likely due to a higher level of travel- and work-related PA [36]. The high levels of PA in Uganda is also in line with a recent national survey [37]. In sub-Saharan Africa, urban regions have been associated with a more sedentary lifestyle which may explain lower self-reported PA in the South African versus Ugandan setting [16]. Besides these global trends, a substantially higher proportion of the Ugandan participants reported to be employed.

Self-reported frequency of vigorous PA showed a similar negative association with HbA1c across the three settings. Regression estimates for self-reported frequency of moderate PA were about half the size of vigorous PA estimates and non-significant. This is not in line with a recent meta-analysis of randomized trials which found changes in HbA1c driven by the duration of PA in a linear manner and independent of the type and intensity of the PA intervention [32]. If only duration and not intensity of PA would matter, a straightforward explanation of our findings would be that participants’ reporting of PA is dependent on the intensity of PA. In other words, participants’ perception to have performed 30 min. of moderate PA may be different from their perception to have performed 15 min. of vigorous PA. These findings from a real-life setting warrant further investigation as they would be crucial to consider in health promotion. Experimental trials using objective and self-reported measures can bring more insight.

### Study limitations and recommendations

Comparison of motivational constructs was possible across settings, but measures had to be modified to obtain adequate measurement models. This incompatibility across very different contexts could be explained by translation to local languages altering certain nuances, a different contextual relevance of certain items and a different understanding by participants from different settings. As mentioned in the results section, two factor loadings of the barrier self-efficacy construct were very low for the Swedish site. However, sensitivity analysis after exclusion of the item with the lowest factor loading did not reveal any major differences.

We further need to acknowledge that sampling procedures and criteria were different in the three settings for pragmatic reasons. As we documented in the methods section, participants in the Ugandan setting were recruited through a random sampling procedure, while participants in the other sites were mainly recruited from health centers, at the cost of reducing external validity. Moreover, selection criteria also differed across sites: participants from the Ugandan and South African site had not been diagnosed with T2D for longer than 12 months, while this was 5 years for the Swedish site. Such differences may have influenced the mean levels and relationships between the motivational constructs in this study. Another limitation of our cross-comparison study was that we did not directly assess relevant cultural differences (e.g. via measures designed to assess individualism/collectivism) which could have been served as possible moderators of the effects we measured.”

The distinction between self-reported vigorous and moderate PA offered an interesting perspective, but also hindered the association of constructs with other factors that determined PA performance. A distinction between categories of PA (e.g., work-, leisure-, transport-related PA) and other categories of motivation from the SDT continuum may add further insight into the role of motivation. In addition, controlling for other factors (e.g. perceived safety, availability of sports infrastructure, etc.) may result in a more nuanced image of the role of motivation.

This study aimed to assess the cross-cultural validity of an SDT process model across different settings. However, the cross-sectional design of this study does not provide evidence for causal pathways or trends over time. While this study focused on people who were recently diagnosed, different dynamics may appear in people with long-standing diabetes. Studies collecting data at different time points and intervention trials can address these shortcomings.

Finally, we need to acknowledge that the use of self-reported measures exposes our findings to bias, including social desirability bias, recall bias (people who value PA more as beneficial for health, may also have reported higher values of PA) and interviewer bias. Shared method variance between measures may have led to overestimation of associations. Objective registration of PA through a pedometer or accelerometer could have made our findings more robust and challenged self-reporting.

## Conclusion

This is the first study comparing a motivational process model between Western and sub-Saharan African settings. Our findings suggest a similar understanding of these constructs across very different settings which makes comparison meaningful. Perceived social support and barrier self-efficacy levels showed to be lower in the urban samples with a high proportion of migrants in South Africa and Sweden, suggesting them to be psychosocially more vulnerable. Except for the association between perceived social support and identified regulation, the motivational process model was different across settings. Identified regulation showed stronger associations with PA outcomes than socials support and self-efficacy. However, we found that these relationships were dependent on the perceived intensity of PA, and hence, do not necessarily reject the etic validity of the underlying behavioral theory. We recommend more specific and objective PA outcomes to analyze these associations. Self-reported vigorous PA was related to lower Hba1C values across the three settings, while this was not the case for moderate PA. This discrepancy urges further exploration of people’s perception of moderate and vigorous PA as it may have major implications for health promotion and education. Our study showed it is feasible to compare a sophisticated motivational model across very different settings. We encourage further research on the cross-sociocultural validation of such models.

## Supplementary Information

**Additional file 1.**

## Data Availability

The datasets used and/or analysed during the current study are available from the corresponding author on reasonable request.

## Abbreviations

BMI: Body mass index
CFA: Confirmatory factor analysis
CFI: Comparative fit index
FINDRISC: Finnish Diabetes Risk Score
Hba1C: Hemoglobin A1c
MGSEM: Multigroup structural equation modelling
PA: Physical activity
RMSEA: Root-mean-square error of approximation
SDT: Self-determination theory
SRMR: Standardized root mean square residual
T2D: Type 2 diabetes
TLI: Tucker-Lewis index
WEIRD: Western, educated, industrialized, rich and democratic
