# Supplementary material for: Motivational determinants of physical activity in disadvantaged populations with (pre)diabetes: a cross-cultural comparison
Source: BMC Public Health. 2022 Jan 24;22:164. doi: 10.1186/s12889-022-12539-9 (PMC8787976; doi:10.1186/s12889-022-12539-9)
Supplement: Supplementary file 1 — Additional file 1. [file 12889_2022_12539_MOESM1_ESM.docx]

**Supplementary File 1: Questionnaire Items and Factor Loadings**

| PERCEIVED SOCIAL SUPPORT: PARTICIPATION OF SIGNIFICANT OTHERS |
| --- |
| Intro: **We want to understand to what extent people close to you (friends, family or relatives) have helped you to do physical activity.** |
| SS 1: How often have people close to you (friends, family or relatives) **exercised with you**? |
| SS 2: How often have people close to you (friends, family or relatives) **encouraged you to exercise**? |
| SS 3: How often have people close to you (friends, family or relatives) **changed their schedule so you could exercise together**? |
| SS 4: How often have people close to you (friends, family or relatives) **discussed exercising with you**? |
| SS 5: How often have people close to you (friends, family or relatives) **helped you with exercising on special occasions such as holidays, feasts, family gatherings**? |
| Answer options:  1= Never  2= less than once a week  3= once a week  4= more than once a week  888= no answer/not applicable |

| BARRIER SELF-EFFICACY |
| --- |
|  |
|  |
| SE 1: Do you think you can be physically active **even during holidays, weddings or other special events**? |
| SE 2: Do you think you can be physically active **even if your family does not encourage you to be physically activity**? |
| SE 3: Do you think you can be physically active **even if you think it is not the best weather for doing sports**? |
| SE 4: Do you think you can be physically active **even if you are in a place with no exercise facilities or safe roads for walking**?  (probing ‘for instance at home or at work or in another way’) |
| SE 5: Do you think you can be physically active **even if you have health problems?** |
| SE 6: Do you think you can be physically active **even if no other people around you are doing exercise or walking**? |

| IDENTIFIED REGULATION |
| --- |
| IR1: Would you do physical exercise **because** **you personally believe it is the best thing for your health**? |
| IR2: Would you do physical exercise **because it is very important for being as healthy as possible**? |
| IR3: Would you do physical exercise **because you feel that you want to take responsibility for your own health**? |
| IR4: Would you do physical exercise **because** **it is an important choice you really want to make**? |
| Answer options:  1 Strongly disagree  2 Disagree  3 Neutral  4 Agree  5 Strongly agree  888 Not applicable |

| BEHAVIOURAL MEASURES – PHYSICAL ACTIVITY |
| --- |
| Please think about the last ONE MONTH and the last SEVEN DAYS when you answer these questions. |
| On how many of the last SEVEN DAYS did you do **vigorous activities** for **at least 15 minutes**, such as cycling uphill or at fast pace; swimming laps; carrying heavy loads; shovelling or digging; jogging; running or a sport? |
| On how many of the last SEVEN DAYS did you do **moderate activities** for **at least 30 minutes**, such as recreational swimming; gardening; heavy cleaning such as washing windows, vacuuming, sweeping or mopping; brisk walking; biking at moderate pace; etc.? |

**Model parameters for Perceived Social Support and Barrier self-efficacy including all questionnaire items.**

|  | **Uganda** |  | **South Africa** | | **Sweden** |  |
| --- | --- | --- | --- | --- | --- | --- |
| **Perceived Social Support** | | |  |  |  |  |
|  | **λ** | **P-value** | **λ** | **P-value** | **λ** | **P-value** |
| SS1 | 0.796 |  | 0.794 |  | 0.738 |  |
| SS2 | 0.793 | 0.000 | 0.805 | 0.000 | 0.685 | 0.000 |
| SS3 | 0.855 | 0.000 | 0.833 | 0.000 | 0.673 | 0.000 |
| SS4 | 0.802 | 0.000 | 0.776 | 0.000 | 0.604 | 0.000 |
| SS5 | 0.650 | 0.000 | 0.726 | 0.000 | 0.605 | 0.000 |
|  |  |  |  |  |  |  |
| **Model fit:** |  |  |  |  |  |  |
| CFI | 0.984 |  | 0.96 |  | 0.927 |  |
| TLI | 1.000 |  | 1.000 |  | 1.000 |  |
| RMSEA | 0.09 |  | 0.148 |  | 0.133 |  |
| 90% CI RMSEA | 0.054 - 0.131 | | 0.104 - 0.196 | | 0.066 - 0.206 | |
| RSMR | 0.022 |  | 0.034 |  | 0.055 |  |
|  |  |  |  |  |  |  |
| **Barrier Self-efficacy** | |  |  |  |  |  |
|  | **λ** | **P-value** | **λ** | **P-value** | **λ** | **P-value** |
| SE1 | 0.54 |  | 0.51 |  | 0.257 |  |
| SE2 | 0.515 | 0.000 | 0.421 | 0.000 | 0.765 | 0.000 |
| SE3 | 0.8 | 0.000 | 0.804 | 0.000 | 0.415 | 0.000 |
| SE4 | 0.712 | 0.000 | 0.793 | 0.000 | 0.599 | 0.000 |
| SE5 | 0.59 | 0.000 | 0.614 | 0.000 | 0.324 | 0.000 |
| SE6 | 0.507 | 0.000 | 0.499 | 0.000 | 0.887 | 0.000 |
|  |  |  |  |  |  |  |
| **Model fit:** |  |  |  |  |  |  |
| CFI | 0.951 |  | 0.824 |  | 0.981 |  |
| TLI | 0.918 |  | 1.000 |  | 1.000 |  |
| RMSEA | 0.088 |  | 0.187 |  | 0.047 |  |
| 90% CI RMSEA | 0.061 - 0.117 | | 0.161 - 0.214 | | 0.000 - 0.114 | |
| RSMR | 0.043 |  | 0.09 |  | 0.047 |  |

*Note:* Factor loadings (λ) are standardized. P-values produced by testing the null hypothesis that the unstandardized factor loading equals zero in the population. SS Social Support; SE Self-efficacy; CFI comparative fit index; TLI Tucker-Lewis index; RMSEA root-mean-square error of approximation; RMSEA 90% CI 90% confidence interval for RMSEA; SRMR standardized root mean square residual

**Model parameters for Perceived Social Support, Barrier self-efficacy and identified regulation after exclusion of items.**

|  | **Uganda** | | **South Africa** | | **Sweden** | |
| --- | --- | --- | --- | --- | --- | --- |
|  | **λ** | **P-value** | **λ** | **P-value** | **λ** | **P-value** |
| **Perceived Social Support** | | | | | | |
| SS1 | 0.772 |  | 0.768 |  | 0.795 |  |
| SS2 | excluded | | | | | |
| SS3 | 0.888 | 0.000 | 0.876 | 0.000 | 0.730 | 0.000 |
| SS4 | 0.782 | 0.000 | 0.731 | 0.000 | 0.495 | 0.000 |
| SS5 | 0.659 | 0.000 | 0.752 | 0.000 | 0.588 | 0.000 |
|  |  |  |  |  |  |  |
| **Barrier Self-efficacy** | | | | | | |
| SE1 | excluded | | | | | |
| SE2 | excluded | | | | | |
| SE3 | 0.813 |  | 0.805 |  | 0.661 |  |
| SE4 | 0.733 | 0.000 | 0.818 | 0.000 | 0.543 | 0.032 |
| SE5 | 0.591 | 0.000 | 0.612 | 0.000 | 0.322 | 0.039 |
| SE6 | 0.506 | 0.000 | 0.460 | 0.000 | 0.232 | 0.115 |
|  |  |  |  |  |  |  |
| **Identified Regulation** | | | | | | |
| IR1 | 0.613 |  | 0.709 |  | 0.608 |  |
| IR2 | 0.635 | 0.000 | 0.748 | 0.000 | 0.681 | 0.002 |
| IR3 | 0.599 | 0.000 | 0.779 | 0.000 | 0.738 | 0.000 |
| IR4 | 0.516 | 0.000 | 0.707 | 0.000 | 0.665 | 0.018 |

*Note:* Factor loadings (λ) are standardized. P-values produced by testing the null hypothesis that the unstandardized factor loading equals zero in the population. SS Social Support; SE Self-efficacy; IR Identified regulation; CFI comparative fit index; TLI Tucker-Lewis index; RMSEA root-mean-square error of approximation; RMSEA 90% CI 90% confidence interval for RMSEA; SRMR standardized root mean square residual
